# Isolated unilateral absence of the pulmonary artery (IUAPA) presenting with progressive interstitial shadows: A case report

**DOI:** 10.1002/rcr2.70048

**Published:** 2024-10-16

**Authors:** Tomohito Takeshige, Ryo Koyama, Makiko Kohmaru, Takashi Akimoto, Junko Watanabe, Toshifumi Yae, Kenji Kido, Kazuhisa Takahashi

**Affiliations:** ^1^ Department of Respiratory Medicine Juntendo University Nerima Hospital Tokyo Japan; ^2^ Department of Respiratory Medicine Juntendo University Faculty of Medicine and Graduate School of Medicine Tokyo Japan

**Keywords:** interstitial change, interstitial lung disease (ILD), interstitial shadow, isolated unilateral absence of the pulmonary artery (IUAPA), unilateral absence of pulmonary artery (UAPA)

## Abstract

Unilateral absence of the pulmonary artery (UAPA) is a rare congenital condition. When UAPA occurs without associated congenital heart disease, it is referred to as isolated unilateral absence of the pulmonary artery (IUAPA). IUAPA is frequently not diagnosed until adulthood. A 61‐year‐old female patient presented with right chest pain and a worsening interstitial shadow over 9 years. Contrast‐enhanced computed tomography revealed the absence of blood flow from the pulmonary artery in the right lung, leading to the diagnosis of IUAPA. In this case, the diagnosis of UAPA was established after approximately 40 years. This case underscores the importance of identifying vascular abnormalities to differentiate this condition in patients presenting with nonspecific symptoms and interstitial shadows.

## Graphical abstract

Here, we present a case of isolated unilateral absence of the pulmonary artery (IUAPA) diagnosed at age 61 due to progressive interstitial changes.

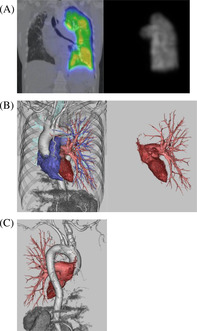

## INTRODUCTION

Unilateral absence of the pulmonary artery (UAPA) is a rare congenital condition, thought to result from a developmental abnormality involving the connection between the 6th branchial arch artery and the pulmonary trunk.^1^ First described by Frantzel in 1868, its prevalence is estimated at approximately 1 in 200,000 individuals. When UAPA occurs without associated congenital heart disease, such as truncus arteriosus, tetralogy of Fallot, or atrial septal defect, it is referred to as isolated unilateral absence of the pulmonary artery (IUAPA). IUAPA accounts for approximately 30% of UAPA cases and is often not diagnosed until adulthood. Interstitial changes in the lungs are observed in 14% of cases.^1^

Here, we present a case of IUAPA diagnosed at age 61 due to progressive interstitial changes.

## CASE REPORT

**FIGURE 1:**
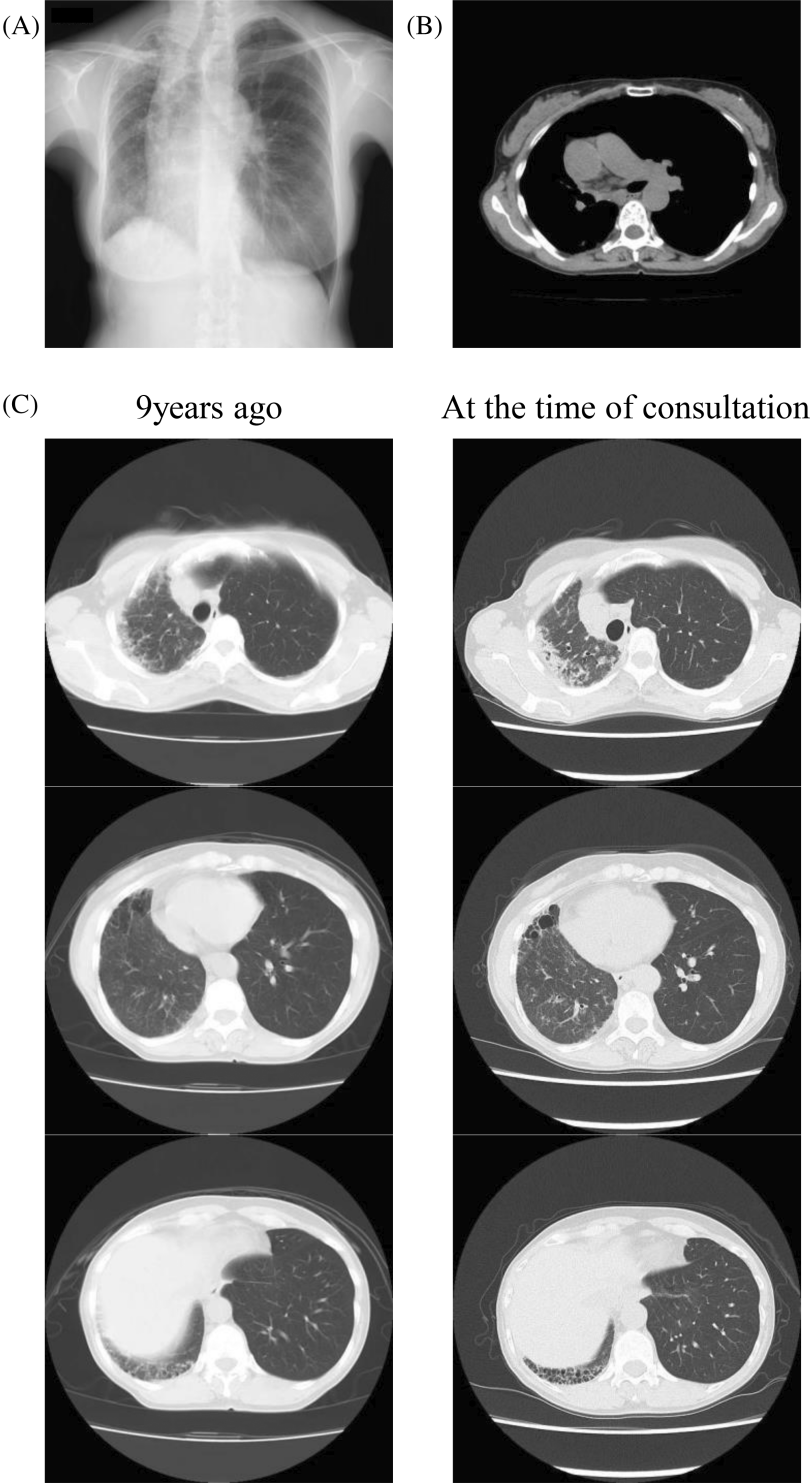
(A) Asymmetry of the thoracic cage with mediastinal shift to the right, ground‐glass opacity, and infiltrative shadow in the upper right lung field, along with reticular shadowing in the right lower lung field. (B) The right pulmonary artery is not visible. (C) Time series of chest computed tomography images: Left column shows images from 9 years ago; right column shows images from the time of consultation. Consolidation in the right upper lobe, cystic changes in the right middle lobe, and honeycombing in the right lower lobe have progressed over time.

**FIGURE 2:**
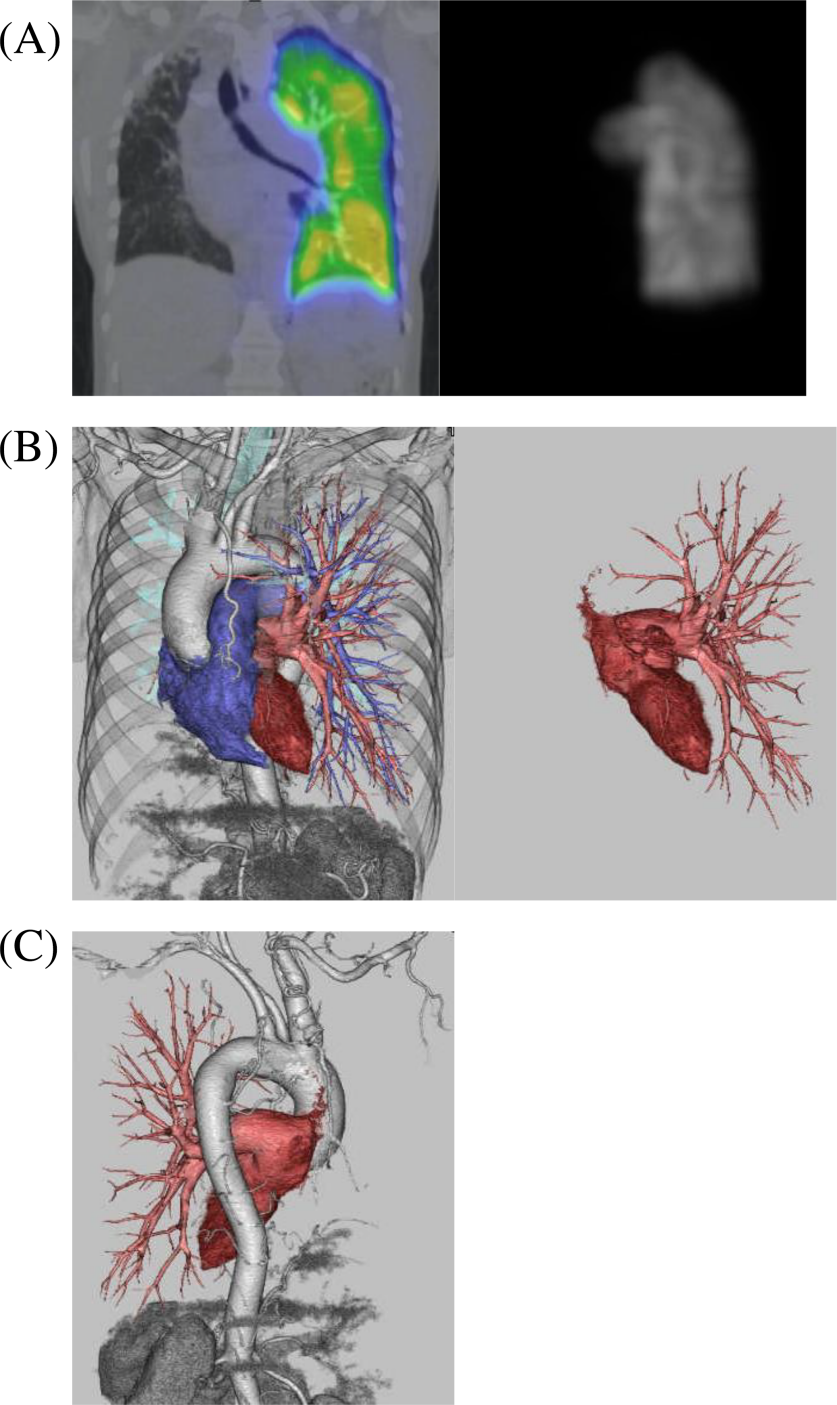
(A) Pulmonary blood flow scintigraphy shows no accumulation in the right lung. (B) Contrast‐enhanced computed tomography images reveal the absence of blood flow from the pulmonary artery in the right lung. (C) Collateral circulation from the bronchial artery to the right lung was observed.

A 61‐year‐old female patient had been monitored for an abnormal chest x‐ray and computed tomography (CT) scan, revealing right lung hypoplasia and an interstitial shadow of unknown origin. She had no history of smoking and no occupational history of dust or asbestos exposure. She presented to our hospital with worsening interstitial shadows over the past 9 years and reported a history of a small right lung diagnosed around age 10. She also presented with right‐sided chest pain. Auscultation revealed fine crackles at the right rear. Chest radiography showed thoracic cage asymmetry, mediastinal shift to the right, ground‐glass opacity, an infiltrative shadow in the upper right lung field, and a reticular shadow in the right lower lung field (Figure 1A). The right pulmonary artery was not visible on chest CT (Figure 1B). Nine years earlier, imaging had shown consolidation in the right upper lobe, cystic changes in the right middle lobe, and honeycombing in the right lower lobe. These changes have progressed over time (Figure 1C). Echocardiography indicated normal cardiac function, and no abnormalities such as pulmonary hypertension or valvular heart disease were found. Respiratory function tests revealed a vital capacity of 2.03 L (74.9% of predicted) and a diffusing capacity of the lung for carbon monoxide of 11.8 mL/min/mmHg, indicating restrictive ventilatory impairment and reduced diffusibility. The Krebs von den Lungen‐6 (KL‐6) level was within normal limits (404 U/mL), while the pulmonary surfactant protein D (SP‐D) level was elevated at 129 ng/mL (normal range: <110 ng/mL). Further investigation with pulmonary perfusion scintigraphy and contrast‐enhanced CT revealed no perfusion in the right lung, and collateral circulation from the bronchial artery to the right lung was observed (Figure 2). In the absence of the right pulmonary artery and with no other cardiovascular abnormalities, the patient was diagnosed with IUAPA. The patient was monitored for pulmonary hypertension, infection, and hemoptysis.

## DISCUSSION

Approximately 85% of IUAPA cases present with symptoms, but diagnosis typically occurs about 5 years after symptom onset. The average age at diagnosis is 40 years.^1^ Common symptoms include hemoptysis, exertional dyspnea, and recurrent respiratory infections. Due to their nonspecific nature, these symptoms often delay diagnosis. In this case, the symptom was chest pain, which was reported in 15.4% of previous cases.^1^ It took approximately 50 years from the initial detection of the abnormality to the diagnosis of IUAPA.

The overall mortality rate for UAPA is 7%. Causes of death include massive pulmonary haemorrhage, right heart failure, respiratory failure, and pulmonary hypertension.^2^ It is crucial to monitor for these complications throughout the disease course.

There are no established treatments or guidelines for IUAPA. Treatment recommendations are based on various case studies. Patients without cardiopulmonary function problems or few or no symptoms do not require further treatment. Periodic cardiac ultrasound is recommended to monitor the progression of pulmonary hypertension. Surgical, interventional, and medical management should be considered for symptomatic patients with exertional dyspnea, edema, hemoptysis, or recurrent respiratory tract infections. Surgical management includes lung resection, revascularization, aortopulmonary shunt, and heart‐lung transplantation. Interventional management includes selective embolization of the collateral arteries and pulmonary angioplasty. Medical management includes the management of right heart failure due to severe pulmonary hypertension or other supportive treatments for respiratory infections.^3^

Interstitial lung changes occur in 14% of cases, with hypoperfusion hypothesized to impair lung parenchyma development, leading to interstitial and cystic changes.^1^ Reduced pulmonary blood flow can also cause bronchiectasis, bullae formation, ciliary dysfunction, sputum retention, and chronic bronchitis, resulting in recurrent respiratory tract infections.^3^ In this case, interstitial and cystic changes progressed over a 9‐year follow‐up period. A review of previous literature did not reveal any cases documenting the long‐term natural history of interstitial changes using chest CT. Therefore, identifying vascular abnormalities is essential for differentiating the disease in patients with nonspecific symptoms and interstitial shadows.

KL‐6 and SP‐D have been reported to be highly specific serum markers for interstitial lung disease (ILD).^4^ In this case, the SP‐D level was elevated, which was thought to reflect interstitial changes in the lungs.

Decreased pulmonary blood flow in patients with pulmonary fibrosis is associated with increased lung abnormalities, suggesting a link between pulmonary fibrosis progression and reduced pulmonary blood flow.^5^ Although this report focuses on a congenital disease, it highlights the potential importance of vascular abnormalities as a cause of ILD.

## AUTHOR CONTRIBUTIONS

Tomohito Takeshige drafted the manuscript. Tomohito Takeshige, Ryo Koyama, Makiko Kohmaru, Takashi Akimoto, Junko Watanabe, Toshifumi Yae, Kenji Kido, and Kazuhisa Takahashi participated in the diagnosis and treatment of the UAPA. Kazuhisa Takahashi provided treatment‐related advice. All the authors have read and approved the final version of this manuscript.

## FUNDING INFORMATION

No funding was required for this report.

## CONFLICT OF INTEREST STATEMENT

None declared.

## ETHICS STATEMENT

The authors declare that appropriate written informed consent was obtained for the publication of this manuscript and the accompanying images.

## Data Availability

Data sharing is not applicable to this article as no new data were created or analyzed in this study.
